# Moral submissiveness: social origin as a vulnerability for well-being on a warming planet

**DOI:** 10.3389/fpsyg.2024.1355736

**Published:** 2024-03-15

**Authors:** Vanessa Weihgold

**Affiliations:** ^1^International Center for Ethics in Science, University of Tübingen, Tübingen, Germany; ^2^Centre Gilles Gaston Granger, Aix-Marseille-Univerity, Aix-en-Provence, France

**Keywords:** moral submissiveness, climate emotions, social origin, well-being, vulnerability, climate change, agency

## Abstract

In recent years, the emotional experience of climate change has been studied extensively from fields like psychology, theology, sociology, and philosophy. It is crucial to analyze these results for possible vulnerability with regard to well-being. While climate justice research raises awareness of the current (social) situation of the participants in relation to the experience of climate change, the research on climate emotions seems to overlook the participant’s former social situation – their family of origin. Previous studies on injustice have shown however that it is precisely the way people were educated on emotion work that has a significant impact on their experiences and sense of control in the situation. Given the importance of this sense of control for mental well-being, I argue consequently that social origin is a vulnerability for well-being in the (emotional) experience of climate change, perpetuating climate injustice, based on this combination of studies from different epochs. Therefore, in the interest to protect well-being on a warming planet, it is crucial to raise awareness of the impact of social origin.

## 1 Introduction

This paper is a philosophical examinations of social upbringing and its’ influence on the psychological situation and emotional experience of climate change and climate injustice inspired by my reading of Moore’s (1979) *Injustice: the social bases of obedience and revolt*. In this book, Moore argues that a lower position in the social hierarchy is connected to moral submission through social structures and social discourses. Hence, he concludes, that revolt becomes more difficult because of a learned submissiveness to these structures and discourses. While Moore uses the term submission interchangeably for both the submitting power structures and the reaction of the lower classes, I have chosen to differentiate between submission as the act of submitting through social discourse or structural injustice and submissiveness as the reaction through Learned Helplessness. Thus, social power dynamics perpetuate injustice.

The United Nations has included well-being for all ages in the Sustainable Development Goal on health. According to the World Health Organization (WHO), well-being is defined as a positive state that enables individuals “to contribute to the world with a sense of meaning and purpose” (World Health Organization, 2023). As Lynne Friedli points out, this generally also includes positive emotions, cognition, and social relations (Friedli, 2009, 9). Psychology aims to abstract from the evaluation of singular situations and links well-being to a capacity to act (agency) that promotes development (Welzel and Inglehart, 2010). Mental health issues, such as depression, may manifest in a blockage of action (Ehrenberg, 1998). Both studies, the one conducted by Christian Welzel and Ronald Inglehart as well as the one by Alain Ehrenberg, demonstrate a correlation between a sense of agency achieved through actively shaping one’s life on the one hand and feelings of satisfaction and meaning on the other. According to Martin Seligman’s research on Learned Helplessness, an unpredictable environment that cannot be controlled and thereby does not allow for agency, results in depression-like states that leave the research subjects indulging adverse conditions (Seligman, 1972). However, it is important to note that control and agency are not the same thing. Agency, understood as an involvement in the world through practices, includes a sense of control (Yanchar, 2021). Therefore, promoting the well-being of humanity requires empowering individuals to develop their agency, capabilities, and stress resilience. But, as mentioned elsewhere, focusing solely on individuals is inadequate, because, as social animals, human mental health and well-being is heavily influenced by social relations (Kałwak and Weihgold, 2022).

Climate change is commonly known for causing unpredictable and uncontrollable environmental changes, leading to negative consequences for well-being. Recent studies on the issue highlight that climate change particularly impacts the emotional well-being leading to “a chronic fear of environmental doom” (Clayton et al., 2017, 68) or to “grief felt in relation to experienced or anticipated ecological losses” (Cunsolo and Ellis, 2018, 275). This is a particularly concerning issue for young people who, according to a broad study carried out by Hickman and colleagues, are affected by a majority (Hickman et al., 2021). If the WHO aims to promote global well-being, these findings suggest that there are significant issues to be addressed with 84% of the 16- to 26-year-olds being worried about their future. It is important to note that these emotions are based on moral judgments about the missing mitigation of climate change. As noted by Hickman et al., they are prompted by a dissatisfaction with governmental reaction and a feeling of betrayal (Hickman et al., 2021, e817). This also suggests an influence of the social on well-being. As we all share this planet, global warming will affect every human (with vulnerable groups being particularly touched). Therefore, people who feel betrayed call for an ethics of conduct on a warming planet, such as Sustainable Development in its broad understanding (Raworth, 2012).

Climate emotions thus translate the experience of changes in the environmental and social climate. Although important for understanding (mental) health and well-being, findings on climate emotions often neglect social origins. Earlier studies on injustice have identified social origin as a major predictor of the moral and emotional experience (Moore Jr, 1979; Galway et al., 2019). Based on my literature research, I argue, that social origin is a vulnerability in this realm not only because of climate injustices, but also because individuals who have grown up in socially disadvantaged classes and with lower educational status have been shown to react with moral submissiveness to the status quo and will thus deal differently with their emotions than those in socially superior positions. In this article, social origin refers to the social status that a person has had during their upbringing.

Research advances not only through novel insights on new subjects but also by examining a subject through the lens of established or maybe even forgotten theories. Therefore, I have chosen to discuss the current research on climate emotions with results on human reactions to injustice as presented by Moore Jr (1979). The significance of the presented argument should not be overlooked. If social origin affects the way how people experience climate emotions, further research is necessary to understand the implications for concrete lived human experience. This will enable psychology and education to incorporate the findings into prevention and treatment.

As a white researcher from the Global North, I cannot speak for people from the Global South. Therefore, when discussing injustice in this paper, I am addressing those within industrialized capitalist societies in Western Europe. While I have not experienced poverty firsthand, as a part time single mother with strong environmental values and a precarious work contract, I feel that I am in a position to speak on this issue.

To support the argument, that a disadvantaged social origin increases vulnerability to experiencing climate emotions due to the perpetuation of climate injustice through moral submissiveness, we must first demonstrate how social origin affects mental well-being and moral submissiveness to injustice in general. This connection is a necessary condition for the argument. In the second part, we will then discuss the sufficient condition with a specific focus on climate emotions, understood as moral emotions negotiating injustice.

## 2 Moral submissiveness to injustice and its influence on well-being

As previously mentioned, individual well-being is not solely influenced by personal factors, but also by social surroundings. Sociology has a longstanding tradition of studying the impact of social class differences and Diana Kuh and Yoav Ben-Shlomo demonstrated that the socio-economic origin influences adult physical health (Kuh and Ben-Shlomo, 2004). The limited access to medical care during childhood has long-term consequences on adult health. Yet, we must expect national differences since most European countries have assurance coverage for at least the most important treatments, unlike the United States. However, limited economic resources are not only a problem in the US for accessing the best available treatment. Additionally, Helen Niemeyer and Christine Knaevelsrud have demonstrated that the negative effect of a lower social status on physical health can also be applied to mental health. Although people with a lower social status experience a higher prevalence of mental disorders, they are underrepresented in those who receive psychological treatment (Niemeyer and Knaevelsrud, 2023). As reasons for this the researchers cite a lack of access to psychological treatment, especially in countries like the US that lack a free health assurance, but also the shortage of practitioners worldwide, making it time-consuming and challenging to find one. Social reasons, like accepting that mental problems are eligible for treatment, may also contribute. As a result, care for (mental) health is significantly impacted by intersecting injustices and social power imbalances.

In the aftermath of World War II, sociologist Barrington Moore, Jr. posed the question of whether there exists a human sense of justice (Moore Jr, 1979, 45–49). His findings indicate that all humans respond to perceived injustices, but individuals with a better education, higher self-esteem, and social status are more likely to get angry and take action, while those with lower education, self-esteem, and social status, are more likely to accept the situation as a given and submit to it. Moore, Jr. links a low social status to low self-esteem because he sees a prevailing social discourse that maintains the existing hierarchy. For instance, in his example of the Untouchables in India, we can observe how religious and social rules are not only followed, but also enforced by the Untouchables themselves on individuals from other backgrounds who question these rules, because the former believe in karma (Moore Jr, 1979, 56–57). Moore, Jr. deduces that suffering has a moral authority (Moore Jr, 1979, 64) and he concludes that moral outrage, i.e., the emotional experience of other people’s infringement of rights and privileges (Goodenough, 1997), is an experience of people with a higher status. In other words, individuals with a lower social status may develop Learned Helplessness in Seligman’s sense, and attribute a moral value to their circumstances. Conversely, those with a higher social status learn that their involvement in the world can affect change and thus experience agency. Individuals from the lower socioeconimic classes not only submit to the injustices but they are also more likely to suppress their emotions. According to Arlie Hochschild, this is due to the fact that lower status jobs often require greater control and emotional labor (e.g., when handling explosives), while decision makers have the privilege of taking their emotions and values into account (Hochschild, 2012, 153–56). Moral submissiveness and emotion work may be considered as coping mechanisms when individuals are unable to respond to unjust situations by other means. However, individuals from socially disadvantaged backgrounds are more likely to experience additional injustices. Therefore, I argue that there is a vicious circle of structural social disempowerment and moral submissiveness perpetuated by Learned Helplessness, which reinforces injustice.

Moreover, research in psychology by Michael Kraus and colleagues has shown that the social class shapes people’s experience of social interactions in general with “potentially far-reaching consequences for overall health and well-being, particularly for people of lower social class.” (Kraus et al., 2011, 1,385) Most importantly, this paper highlights that individuals from lower social ranks are more likely to perceive situations as hostile than their higher-ranking counterparts. Combining these findings with the information presented earlier, it can be concluded that individuals in lower social classes must engage in even more intense emotion work to regulate their emotions. This is because on the one hand, they learned to suppress their emotions while, on the other, perceiving situations as more hostile. Therefore, it can be inferred that individuals with a lower social status constantly experience higher levels of tension and pressure.

All of these examples demonstrate an awareness in the research subjects of their agency in the particular situation they are discussing. Agency, as defined by Stephen Yanchar involves engagement in the world through practices (Yanchar, 2021) that are influenced by past events and oriented toward the future, as noted by Emirbayer and Mische (1998, 962). However, the research cited above primarily refers to individuals’s current situation, even though these are informed by past experiences. Moore, Jr.’s example of the Indian Untouchables, who submit to the idea of being dirty and will step out of the way of people from other castes (Moore Jr, 1979, 54–55), illustrates how a lack of agency informed by past experiences and a prospective future that remains unchanged is turned into an acceptance of and submissiveness to the social rules inflicted upon these people, perpetuating injustice.

In support of this, there is evidence to suggest that upbringing can influence one’s ways of experiencing. As Arlie Hochschild points out, the family serves as the “training ground” (Hochschild, 2012, 156) for future work environments. With reference to sociolinguist Basil Bernstein’s research results^1^, Hochschild points out that a family culture that uses restricted code, such as ‘You have to do this because I am your father and I say so!’, prepares the child for a work environment in which they will have to follow rules. On the other hand, an elaborate family code, such as ‘Could you please close the door, I am freezing.’, persuades the child to choose the ‘right’ action (Hochschild, 2012, 157), thereby creating agency and preparing them to take on responsibilities in the workplace. Although there is still a (social) expectation for the child to comply with the parent’s request in the latter case, the child is provided with more information and addressed in a way that acknowledges their empathetic abilities. This approach does not only foster sympathy but also a sense of agency, which can both be limited when a child is given rigid orders to follow and expected to adhere to a strict hierarchy.

The flexibility of the family hierarchies is not solely a question of family culture. Sturctural contingencies that limit families with lower income also play a role, as previously mentioned in relation to accessing medical help. Research has shown that parents who work non-standard times and/or several jobs to support their family, and might still struggle to provide enough food, experience feelings of overwork and stress that directly impact their children’s well-being (Strazdins et al., 2004; Hudson, 2016). When parents are forced to be away for extended periods of time, they are unabled to create a secure bonding environment for their children. The attachment with parents and other significant adults, however, creates a sense of security that is important for developing the ability to cope with adversities in adulthood, as has been shown by Rasmussen et al. (2019). Children learn by example how to deal with stress and insecurity, and how their parents are handling the injustice. Therefore, they will require support from other sources to learn how to depend on presence of others for support.

Although, there is a strong correlation between the social origin and adult social status, it important to note that this is not predetermined. Not only are there examples of parents living in poverty who manage very well to provide a safe environment for their children, but there are also other adults and attachment figures who play a significant role in a child’s well-being. As a result, it is important to understand these research findings as general tendencies (vulnerabilities) rather than as causal links.

In summary, research from psychology, sociology, and linguistics indicates that social status and social origin influence how individuals perceive their agency, which is an indicator of well-being. Those who grew up and lived in a higher-ranking social class are more likely to feel moral outrage when confronted with injustice and take action, while those from lower-ranking social classes tend toward moral submissiveness and may not act. Considering that the perceived agency is correlated with well-being, it can be concluded that the social origin is a vulnerability when facing injustice. As we live on a warming planet, we will now apply these findings to climate emotions. To do so, we have to first establish a link between climate emotions and justice.

## 3 Social origin as vulnerability for climate emotions

Regarding climate change, research on climate justice alerts to the increased vulnerability of people(s) and communities with lower social status who are exposed to a greater number of risk factors, such as raising sea levels, droughts, and air pollution, which also affect their health (Agyeman et al., 2007; Whyte, 2017; Bhavnani et al., 2019; Roberts-Gregory, 2021; Sultana, 2022). Vandana Shiva notes, that children and women are particularly vulnerable within these groups (Shiva, 2014). The American Psychological Association has observed a link between physical health and chronic stress caused by climatic changes (Clayton et al., 2017, 23–24). The intersections, in the sense of interactive constituation and interlocked character of multiple injustices (Cho et al., 2013, 787), of living in a geographic region that experiences severe impacts of global warming, having lower income or communal resources, and being part of a socially suppressed population (such as Indigenous, Black, or disabled individuals) lead to a higher level of stress in the face of climate change (Clayton et al., 2017, 31). While these correlations certainly cannot be generalized or even understood as causal, the intersecting vulnerabilities of this part of this population segment have a greater impact on physical health and well-being because stress in the face of climate change affects them more heavily than it does socially dominant groups. As previously mentioned, it can be expected that poorer citizens have less resources to respond to these impacts. However, it is needless to say, that there are examples of movements from disadvantaged social groups and minorities that have defied injustices and climate change (e.g., Méndez, 2018).

As previously stated, research on climate emotions has thus far overlooked the social origins of their participants. However, there are differences in their capacities to respond to climate injustices, whether financial or other. As a result, the emotional experience of climate change vary necessarily as well. Climate emotions are here defined, with allusion to Despret (2022, 324), as a negotiation of the personal relationship with the social and environmental climate. Climate change has widespread normative implications for individuals and political leaders that concern environmental ethics, but also climate justice. Consequently, climate emotions have a distinct moral character, as they involve the emotional negotiation of injustice. Therefore, I argue that an individual’s social origin can also impact their likelihood for experiencing climate emotions and their resulting sense of well-being, based on their perceived agency or lack thereof.

As discussed, there are various emotions that arise when confronted with (information on) climate change and environmental disruption, such as eco-anxiety, environmental grief or betrayal.^2^ Negotiating the relation with the social and environmental climate including their normative implications, these emotions can be considered as moral emotions. The definition of moral emotions is broad. While Anthony Steinbock defines them as interpersonal feeling experiences (Steinbock, 2014, 12), Florian Cova et al. list five senses of the concept for clarification: emotions that (1) are directed toward a moral object, (2) increase knowledge about moral facts, (3) are a motivation for moral action, (4) foster the moral standards in society or individuals, or (5) are subject to moral assessment (Cova et al., 2015). Regarding climate emotions, all of these senses of the concept apply. They are (1) directed toward promoting a sustainable lifestyle within an unsustainable society, (2) increasing knowledge about climate change^3^ and Sustainable Development, (3) motivating to mitigate climate change, (4) fostering societal responsibility for Sustainable Development, and (5) are highly morally charged, which can lead to feelings of judgment and shame in others, as Budziszewska and Kałwak (2021) and Neckel and Hasenfratz (2021) have shown.

These understandings of moral emotions can be applied to the research on climate emotions. One striking example might be the work of Hickman and colleagues who discuss the feelings of betrayal by politics among young people (Hickman et al., 2021). This concerns the moral object of generational justice, which was already anticipated by Hans Jonas. Jonas called for future generations to be taken into account when discussing the permanence of human life on Earth (Jonas, 1984, 36). Hickman’s research participants have come to the realization that they will experience climate change more severely than their parents, which will limit their life choices. This is a ristriction the older generations did not face.

Similarly, climate anxiety is rooted in the concern for the right to live and the injustice of younger generations facing an uncertain future. The theoretical presupposition for this is evolutionary theory and the drive for survival. Especially psychoanalytical papers such as Hickman et al.’s discuss this as a normal reaction in this context (Hickman et al., 2021, e863). The statement presupposes that climate change is a threat to human survival, eliciting strong emotional responses that are uncontrollable and often subconscious. Therefore, it is inferred that climate emotions reflect the innate sense to preserve human life, creating a societal responsibility to protect life on Earth.

When discussing environmental grief, the connection with a moral object may not be as apparent, but it is still present. There is also a very strong claim for justice in extending grievability, a term coined by Judith Butler, to the more-than-human world, as does Cunsolo (2012). According to this claim, the hierarchies established by Western cultures^4^ have established between human and non-human animals as well as the non-animal environment are not justified within a biocentric worldview. This means that, unlike Jonas, who maintained an anthropocentric approach to the question of responsibility and justice, Cunsolo places life at the center of her theory. It may be debatable when applied generally, but for the case of environmental grief it seems justified as the author has worked with Indigenous peoples who have a different worldview and subsequently different feelings about it than those who have been brought up in a Western culture.

According to this argument, climate emotions can be considered moral emotions that negotiate the injustices of our social and environmental climate. These emotions are related to hierarchies and power imbalances based on factors such as age (generational justice), (economic) status (individual, regional, and national), and species membership. As with other justice-related issues we discussed above, climate emotions should induce either moral outrage or moral submissiveness. This is the case for climate activism that is not only justified by reason, but also with allusion to outrage, as shown by Antadze (2020). Psychological professionals tend to “prescribe” activism because it raises agency and thereby individual resilience (Kałwak and Weihgold, 2022, 6). At the same time, climate inaction is often attributed to conformity with the socially displayed status quo and “fear of becoming an outsider,” as explained by Norgaard (2011, 97). Without knowledge about the social origin of Norgaard’s research participants, it is possible that this is an expression of maintaining their privileges. However, it is argued here that the issue may not solely be rooted in fear, but rather in a long-term experience of lacking agency to change their status (Learned Helplessness (Seligman, 1972)). Individuals from less privileged social backgrounds may internalize a lower sense of agency while growing up, making it more likely for them to submit to their circumstances and morally justify and perpetuate the injustice they face. In this case, social origin could be a vulnerability for experiencing climate emotions or even denialism since individuals may be less resilient and ill-equipped to deal with the situation of climate change.

A possible counterargument may be that this type of reasoning is subject to similar issues as Marxist determinism of social structures: there can be no change and a person’s future social status is always predetermined by their origin. However, there are examples of climate movements, such as those in Bolivia and Ecuador, that originated from Indigenous communities historically of lower social status than the colonizers. These movements gained enough importance as to influence a change in the constitution of their respective countries (Acosta, 2017). As with other grassroots social movements, such as the civil rights movement in the US in the 1950s and 60s, lower social classes may eventually fight for equal rights. However, it is important to note that this does not imply causality, but rather a tendency to moral submissiveness and acceptance of the status quo. Another point to consider is that these movements are socially organized, while submissiveness to an unjust situation often leads to atomization of the social group, as Moore, Jr. points out (Moore Jr, 1979, 65). Therefore, if a social group with a lower status can overcome social submission and cooperate, they have the potential to change the social norms.

To summarize, we have argued that climate emotions are ultimately about justice, particularly in relation to generation and species. Therefore, the findings presented in the previous section on how individuals from diverse social origins address issues of injustice, should also apply to climate emotions. Consequently, individuals with a higher social status during childhood are more likely to exhibit moral outrage and activism, while those a lower social origin tend to display moral submissiveness and inaction. This conclusion is concerning because individuals who benefit from their higher status in the hierarchy may be less likely to act against injustice, as doing so could put them at disadvantage. Moral submissiveness and inaction leading to reduced agency and eventually to forms of depression on the other hand could make individuals vulnerable to climate emotions. It is important to conduct more (empirical) research to determine the relevance of this argumentation for lived experiences. This will enable psychological professionals to take appropriate measures.

## 4 Conclusion

The present article argued that individuals from a disadvantaged social origin may be more vulnerable to experiencing climate emotions, due to their increased likelihood of moral submissiveness to injustice that perpetuates unjust situations. To demonstrate this, we have first investigated research on the influence of social class and origin on (mental) health and well-being in general. We focused then on Barrington Moore, Jr.’s research, which revealed how injustice is maintained through the acceptance of and submissiveness to the prevalent social discourse, even though one is subject to its injustice.

In a second part we considered the implications of the current debate of climate emotions. Negotiating the implications of global warming on the changes in the social and natural climate, these emotions have been shown to have a moral character. Additionally, drawing on results from the climate justice debate, we can assume that low social status intersects not only with physical health risks due to climate change but also with vulnerabilities to well-being. Based on these findings, it can be inferred that individuals with a lower social origin may be more susceptible to submit to the injustices of climate change, further exacerbating their vulnerability.

These findings are important, because they support the need for more psychological and sociological research on the subject of climate emotions. If a disadvantaged social origin increases vulnerability to climate emotions, it should be studied more closely to identify ways to mitigate the impact on well-being, in line with the Sustainable Development Goal on Health and Well-Being.

## Author contributions

VW: Conceptualization, Writing – original draft, Writing – review & editing.

## Footnotes

^1^I am aware, that Bernstein’s wording and worldview are highly debatable and I do not subscribe to his statement that lower classes are poorer in language, but I still think that Hochschild’s point is valid, when applying Bernstein’s findings, that a family who uses an elaborate code will create a different sense of agency in their children than a family who uses a restricted code.

^2^There is a broad variety of climate emotions that is discussed in literature. The present paper has consciously restricted the number of concepts discussed for more clarity.

^3^Following Thomas Potthast, climate change can count as an epistemic-moral hybrid (Potthast, 2014).

^4^The author is aware of the critique that has been addressed at the concept of culture, but since this paper is written from a Global North perspective, I have chosen to keep the term.
